# 5-ALA mediated photodynamic therapy induces autophagic cell death via AMP-activated protein kinase

**DOI:** 10.1186/1476-4598-9-91

**Published:** 2010-04-28

**Authors:** Hong-Tai Ji, Li-Ting Chien, Yu-Hsin Lin, Hsiung-Fei Chien, Chin-Tin Chen

**Affiliations:** 1 Department of Biochemical Science and Technology, National Taiwan University, Taipei 106, Taiwan; 2 Center for Optoelectronic Biomedicine, National Taiwan University College of Medicine, Taipei 100, Taiwan; 3 Department of Surgery, National Taiwan University College of Medicine, Taipei 100, Taiwan

## Abstract

Photodynamic therapy (PDT) has been developed as an anticancer treatment, which is based on the tumor-specific accumulation of a photosensitizer that induces cell death after irradiation of light with a specific wavelength. Depending on the subcellular localization of the photosensitizer, PDT could trigger various signal transduction cascades and induce cell death such as apoptosis, autophagy, and necrosis. In this study, we report that both AMP-activated protein kinase (AMPK) and mitogen-activated protein kinase (MAPK) signaling cascades are activated following 5-aminolevulinic acid (ALA)-mediated PDT in both PC12 and CL1-0 cells. Although the activities of caspase-9 and -3 are elevated, the caspase inhibitor zVAD-fmk did not protect cells against ALA-PDT-induced cell death. Instead, autophagic cell death was found in PC12 and CL1-0 cells treated with ALA-PDT. Most importantly, we report here for the first time that it is the activation of AMPK, but not MAPKs that plays a crucial role in mediating autophagic cell death induced by ALA-PDT. This novel observation indicates that the AMPK pathway play an important role in ALA-PDT-induced autophagy.

## Introduction

Photodynamic therapy (PDT) has been developed as a modality for cancer treatment which combines the use of low energy light with the photosensitizer [1]. The rapid tumor ablation by PDT involves direct cell killings as well as damage to the exposed microvasculature [2]. Singlet oxygen as well as other reactive oxygen species are the major cytotoxic agents responsible for the PDT-induced cellular damages [3]. Cellular and molecular mechanisms involved in PDT-mediated oxidative stress are becoming clear [4,5]. However, the signaling pathways involved in the PDT-mediated cell death are not completely understood.

5-aminolevulinic acid (ALA) itself is not a photosensitizer and serves as the biological precursor in the heme biosynthetic pathway. Exogenous ALA administration leads to the accumulation of PpIX in the mitochondria, which causes direct mitochondrial damage and subsequent cell death after light irradiation [6]. The integration of a complex signaling network results in either cellular repair/recovery or death. Activation of the three major mitogen activated protein kinases (MAPKs), the extracellular signal regulated kinase (ERK), c-Jun N-terminal kinase (JNK), and the p38 kinase have been found in PDT-treated cells [4,5]. Nevertheless, the roles of MAPKs in mediating PDT-induced cell death depend on the cell line and/or photosensitizer used.

AMP-activated protein kinase (AMPK) is a highly conserved heterotrimeric serine/threonine protein kinase that regulates energy homeostasis in mammalian cells [7,8]. It can be activated by ATP depletion or AMP elevation [9]. The activated AMPK can restore energy homeostasis inside cells by inhibiting anabolic reactions and stimulating energy-producing catabolic pathways. Cancer cells exhibit characteristic metabolic demands that are different from normal cells. Being a key metabolic regulator, AMPK may regulate the switch. Other than a metabolic sensor, AMPK also plays a critical role in response to cellular stress such as hypoxia or oxidative stress [10]. In addition, activation of AMPK has been implicated in the regulation of anti-apoptotic [11-13] as well as pro-apoptotic effects [14-16]. Recent studies also indicate the involvement of AMPK in autophagy induced by stimuli such as hypoxia or nutrient-free medium [17-19]. These observations imply that AMPK may be a potential target for cancer treatment.

Autophagy is involved in removing damaged organelles, a process that is required for the promotion of cellular survival during nutrient starvation, pathogen infection, aging, and neurodegenerative processes [20,21]. However, the constitutive activation of autophagy can lead to cell death as a result of excessive self-destruction of cellular organelles [22]. PDT-treated cells can undergo apoptotic or nonapoptotic pathways, depending on the cell type, the photosensitizer, and the PDT dose [5,23]. Recently, PDT-induced autophagy has also been described [5,24]. However, the signaling molecule involved in PDT-induced autophagy is not clear.

Previously, we have shown that ALA mediated PDT could disrupt mitochondrial membrane potential, deplete cellular ATP and hence, cause mitochondrial dysfunction [25]. Since autophagy is a common response to mitochondrial dysfunction [26], we reasoned that ALA-PDT may induce autophagic cell death by interfering with cellular bioenergetics. In this study, we demonstrate that ALA-PDT can trigger autophagic cell death by the regulation of AMPK. Our results indicate that AMPK activation is required for ALA-PDT induced autophagic cell death.

## Results

### Cell death induced by ALA-PDT involves the activation of AMP-activated protein kinase

**Figure 1:**
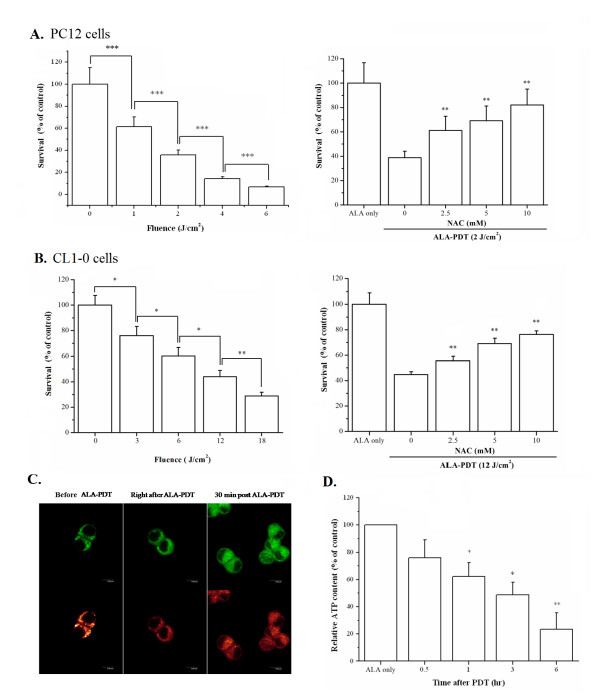
**ALA-PDT-induced oxidative stress results in mitochondrial dysfunction and cell death**. Survival rate of PC12 cells (**A**) and CL1-0 cells (**B**) after ALA-PDT. The right panel shows the cell survival rate of PC12 and CL1-0 cells which were incubated with 1 mM ALA for 3 hr, and then irradiated with different light dose. The left panel shows the survival rate of PC12 or CL1-0 cells pre-incubated with different concentrations of *N*-acetylcysteine (NAC) before ALA-PDT (1 mM ALA, 2 J/cm^2 ^and 12 J/cm^2 ^of light for PC12 and CL1-0 cells, respectively). Cell survival was assessed by Trypan Blue exclusion assay 24 hours after light irradiation. Data were obtained from three independent experiments and are shown as mean ± SD. Significantly different from ALA only; *P < 0.05, **P < 0.01, ***P < 0.001. (**C**) Mitochondrial membrane potential decreased following ALA-PDT in PC12 cells. JC-1 labeling of the cells treated with ALA only (left panel), right after (middle panel) or 30 minutes post light irradiation (right panel) under the light dose of 2 J/cm^2 ^are shown. The upper panel represents the JC-1 monomer (green) and lower panel represents the JC-1 aggregate (red). (**D**) Following ALA-PDT (1 mM ALA, 2 J/cm^2^), PC12 cells lysates were prepared at the indicated times and the ATP contents were measured using an ATP determination kit. The figure is a representative of three independent experiments and the results are shown as mean ± SD. Significantly different from ALA only; *P < 0.05, **P < 0.01.

The effect of ALA-PDT was first examined in the PC12 cell, a cell line derived from the pheochromocytoma cells of the rat adrenal medulla [27]. ALA-PDT induced a significant cytotoxicity in PC12 cells in a light-dose dependent manner as indicated by the Trypan Blue exclusion method (Fig. 1A, left panel). A sub-lethal dose of the light (2 J/cm^2^) which led to ~60% cytotoxicity was chosen for all the following experiments unless otherwise specified. The cytotoxic effect of ALA-PDT could be effectively blocked in a dose-dependent manner by the addition of *N*-acetylcysteine (NAC), a known cell permeable antioxidant (Fig. 1A, right panel). This suggests that the phototoxicity was caused by PDT-induced oxidative stress. Similar results were also found in CL1-0 cells, a human lung adenocarcinoma cell line [28] that is genetically distinct from PC12 cells (Fig. 1B). The mitochondrial integrity was further examined because ALA-PDT induced cytotoxicity has been related to mitochondrial damage in several different cell lines [25,29,30]. As shown in Fig. 1C, loss of mitochondrial transmembrane potential (Δψ_m_) was found in PC12 cells after ALA-PDT. Meanwhile, the mitochondrial ATP content decreased 24% 30 minutes after ALA-PDT, and further diminished by 77% 6 hours after PDT (Fig. 1D). These results indicate that ALA-PDT-induced cell death is related to mitochondrial photodamage.

**Figure 2:**
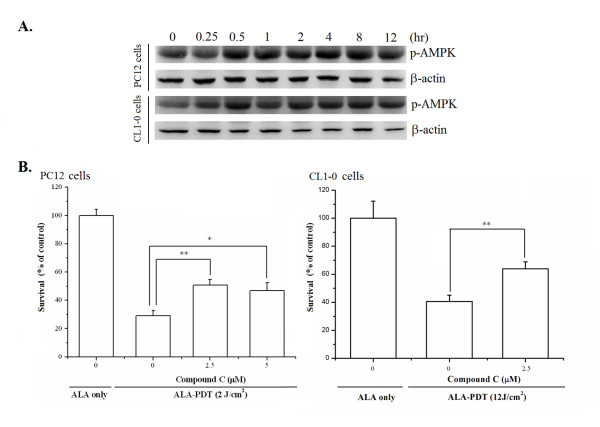
**ALA-PDT induced cell death is mediated through the activation of AMP-activated protein kinase**. (**A**) Western blot analysis of phosphorylated AMPK (p-AMPK) expression in PC12 and CL1-0 cells treated with ALA-PDT under the light dose of 2 and 12 J/cm^2^, respectively. Equal amounts of protein (50 μg) from ALA-PDT-treated cells were loaded at each lane as confirmed by β-actin level. (**B**) Involvement of AMPK in the ALA-PDT mediated cell death. PC12 (left panel) and CL1-0 (right panel) cells were incubated with 1 mM ALA for 3 hr in the presence of different concentration of Compound C (AMPK inhibitor) for 30 min before light irradiation. Cellular cytotoxicity was determined using Trypan Blue exclusion assay. Values are expressed as a percentage relative to those obtained with cells without Compound C treatment. The figure is a representative of three independent experiments and the results are shown as mean ± SD. Significantly different from ALA only; *P < 0.05, **P < 0.01.

As depletion of ATP activates AMPK, we next examined the activity of AMPK following ALA-PDT. The activity of AMPK was monitored with an antibody recognizing the Thr172-phosphorylated AMPK. As shown in Fig. 2A, a rapid elevation of the AMPK activity was found in PC12 cells 30 minutes after light irradiation. Activation of AMPK was also found in CL1-0 cells following ALA-PDT under a light dose that reduced the CL1-0 cell survival by ~60% (12 J/cm^2^) (Fig. 2A). Meanwhile, pretreatment with the specific inhibitor of AMPK (Compound C) [31] rescued both PC12 and CL1-0 cells after ALA-PDT (Fig. 2B). Together, these data indicate that the activation of AMPK is involved in ALA-PDT-induced cell death.

### ALA-PDT induces caspase-independent cell death

**Figure 3:**
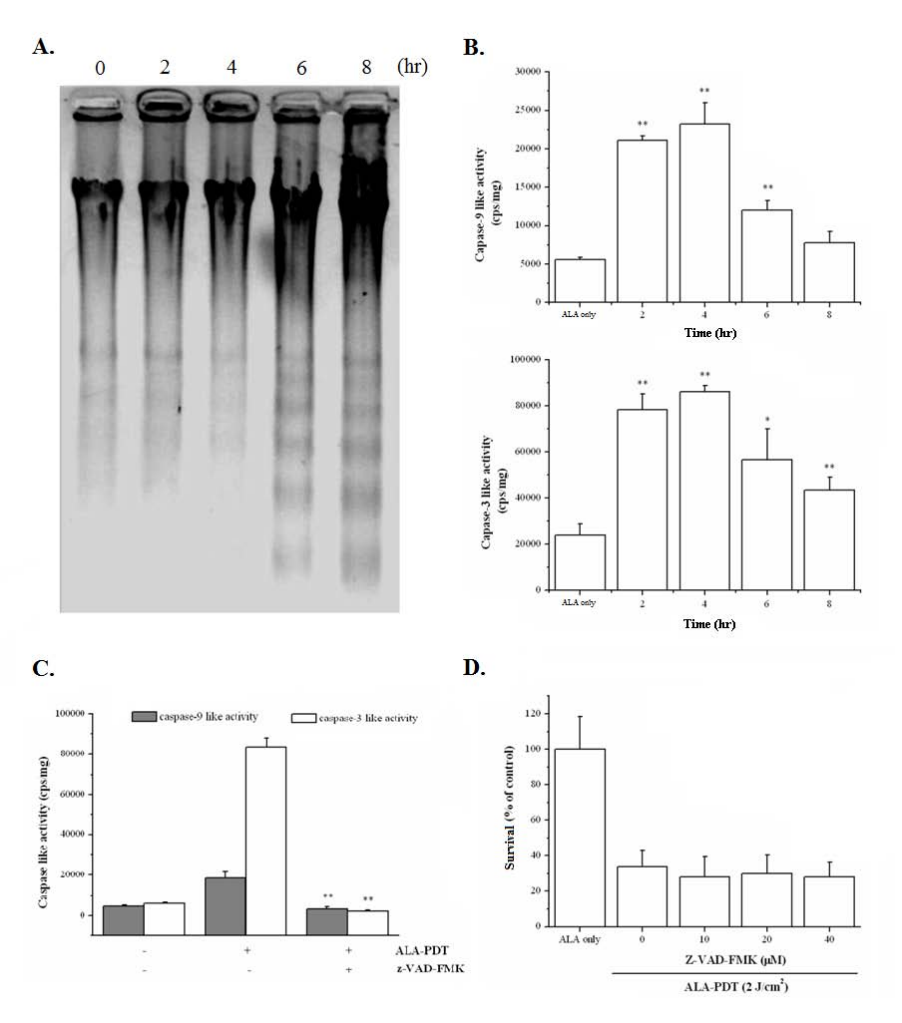
**ALA-PDT induces caspase-independent cell death**. (**A**) PC12 cells were collected at various time points after ALA-PDT (1 mM ALA & 2 J/cm^2^). DNA fragmentation was analyzed by agarose gel electrophoresis followed by ethidium bromide staining. (**B**) PC12 cell lysates were collected at various time points after ALA-PDT (1 mM ALA & 2 J/cm^2^). Caspase-9 (upper panel) and caspase-3 (lower panel) enzymatic activities were analyzed by using Caspase-3/CPP32 and Caspase-9 fluorometric assay kits, respectively. (**C**) After pre-incubation with the pan-apoptotic inhibitor, zVAD-fmk (40 μM), cell lysates were collected at 4 hr post ALA-PDT. Caspase-9 and -3 activities were analyzed. Results are representative of three independent experiments and shown as mean ± SD. (**D**) Cell survival after ALA-PDT is independent of zVAD-fmk dosage. Results are representative of three independent experiments and shown as mean ± SD. No significant different from ALA-PDT only.

It has been largely documented that PDT-induced mitochondrial photodamage may trigger apoptosis, a caspase-dependent cell death pathway [5,23,32]. Therefore, we examined the characteristic indicator of apoptosis, the DNA fragmentation pattern, following ALA-PDT. As shown in Fig. 3A, oligonucleosomal DNA fragmentation was detected as early as 6 hours post ALA-PDT in PC12 cells. It has been shown that apoptosis related to mitochondrial damage entails the activation of caspases-9 and -3. We next examined the caspase-9 and -3 activities in ALA-PDT-treated cells. As shown in Fig. 3B, optimal activation of both caspase-9 (upper panel) and -3 (lower panel) like protease activities were found to increase by more than 5 fold 4 hours following ALA-PDT. Furthermore, the elevated caspase-like protease activity was inhibited by a pan-apoptotic inhibitor, zVAD-fmk (Fig. 3C). Surprisingly, the irreversible inhibition of caspase activation by zVAD-fmk did not protect PC12 cells from ALA-PDT-induced cell death (Fig. 3D). Similar results were also found in CL1-0 cells (data not shown). These results indicate that ALA-PDT-induced cell death is independent of caspase activation in both PC12 and CL1-0 cells.

### Autophagy is the main cell death mode following ALA-PDT

**Figure 4:**
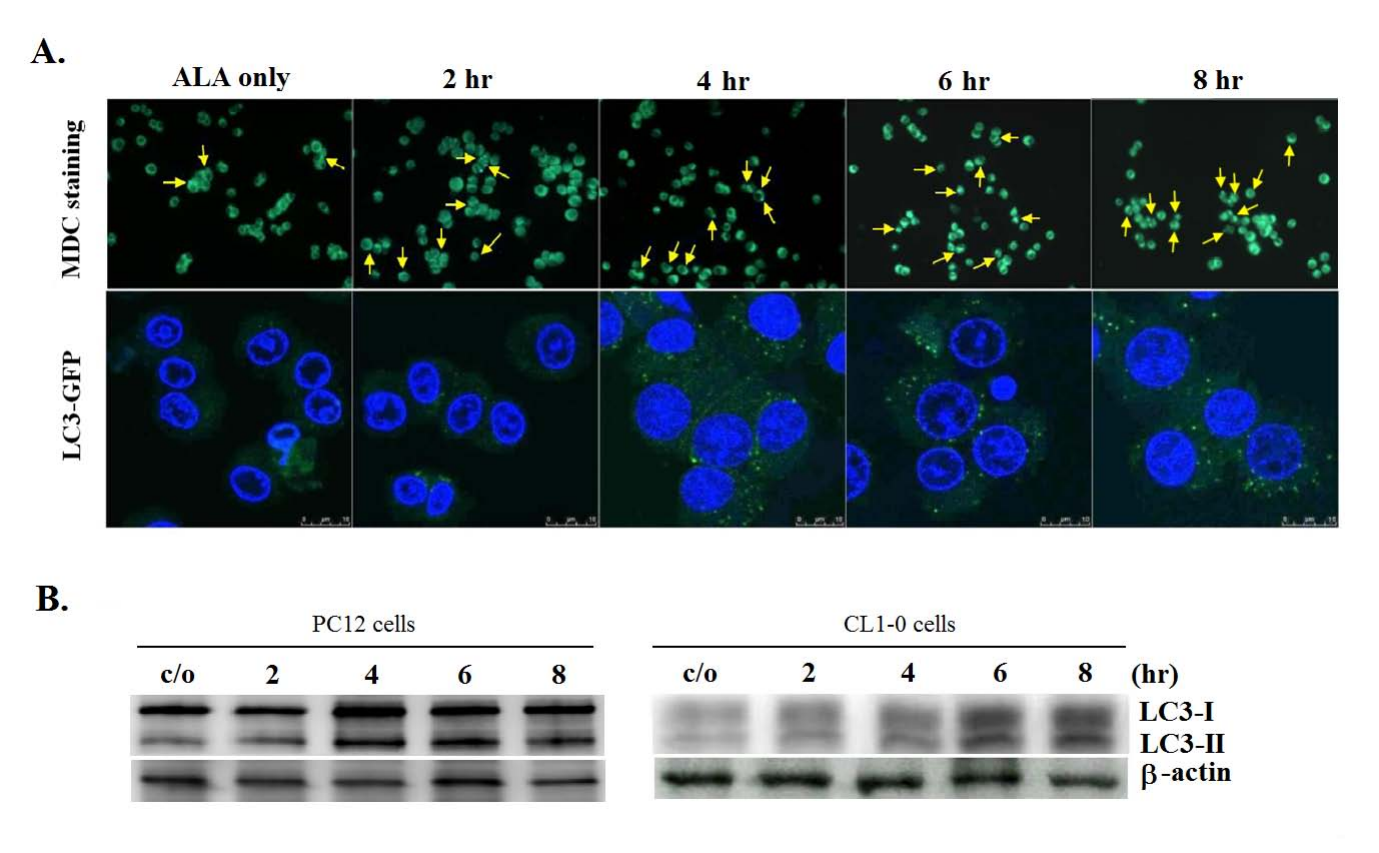
**ALA-PDT induces autophagic cell death in PC12 and CL1-0 cells**. PC12 and CL1-0 cells were treated with 1 mM ALA for 3 hr, and then irradiated under the light dose of 2 and 12 J/cm^2^, respectively. (**A**) Autophagosome staining with MDC (upper panel) and expression pattern of transfected LC3-GFP (lower panel) after ALA-PDT in PC12 cells. In ALA-PDT treated cells, MDC (0.05 mM) accumulated as a punctate pattern (arrows) predominantly in the cytoplasm beginning as early as 2 hr post ALA-PDT. Photomicrographs of MDC staining cells were taken at 200× magnification. LC3-GFP (green), a known autophagosome marker, was expressed around the nuclear region. The nucleus was marked by Hoechst 33342 (blue). (**B**) Immunoblot analysis detected increased levels of the processed LC3-II in PC12 & CL1-0 cells treated with ALA-PDT, but not in cells treated with ALA only (control). β-actin was used as an internal control.

Recently, induction of autophagic cell death has been shown in PDT-treated cells [5,24]. To investigate if autophagy is involved in PC12 cell death following ALA-PDT, cells were incubated with monodansycadaverine (MDC), a fluorescent marker for autophagosomes [33]. As shown in the fluorescence images in the upper panel of Fig. 4A, increased MDC-positive autophagosomes were found as early as 2 hours following ALA-PDT while the control cells showed little MDC incorporation. LC3-GFP, another autophagosome-localizing fusion protein, has facilitated the study of autophagy in cancer cells [34]. The transiently expressed LC3-GFP construct produces diffuse fluorescence in the absence of autophagy and punctuated signals in autophagosomes. In PC12 cells, LC3-GFP began to aggregate in puncta in ALA-PDT-treated cells as early as 2 hours after irradiation, while in control cells the LC3-GFP signal was diffusely distributed in the cytoplasm (Fig. 4A, lower panel).

**Figure 5:**
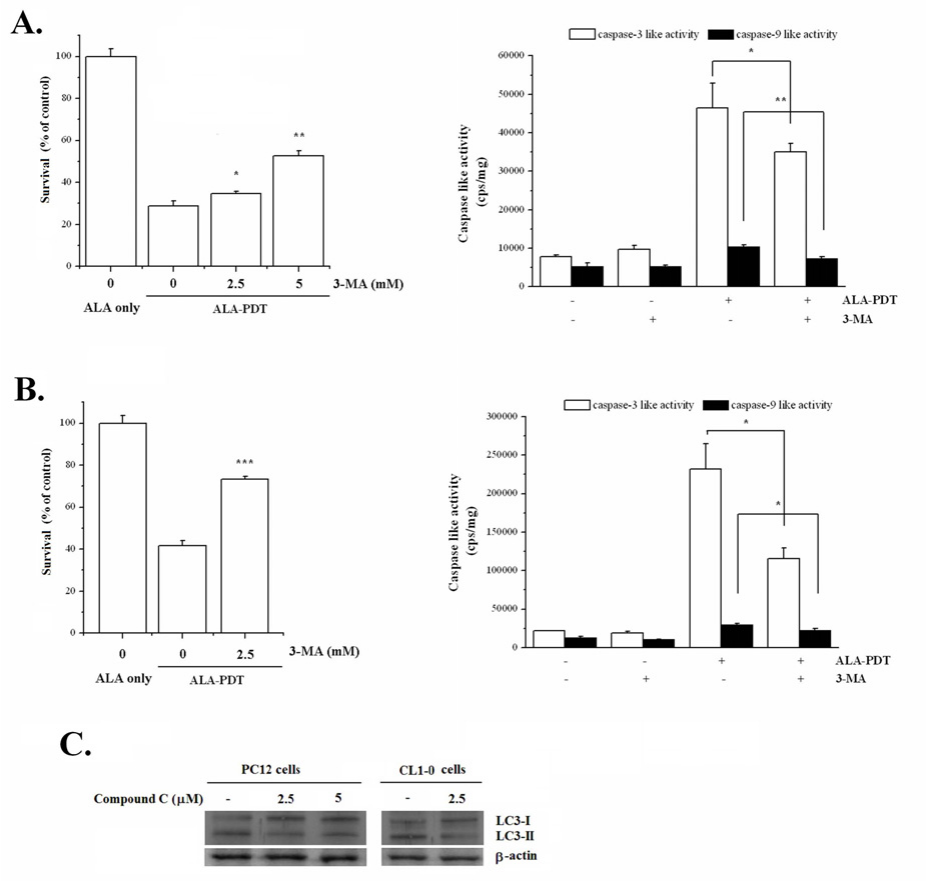
**AMPK mediates autophagic cell death induced by ALA-PDT**. Effects of autophagy inhibitor, 3-MA, on ALA-PDT mediated cell death and caspase activity in PC12 (**A**) and CL1-0 (**B**) cells. PC12 and CL1-0 cells were pre-treated with different concentration of 3-MA before ALA-PDT. The light dose for PC12 and CL1-0 cells were 2 and 12 J/cm^2^, respectively. Cell survival was analyzed 24 hr post ALA-PDT, and the caspase activity was analyzed 4 hr post ALA-PDT. (**C**) Expression level of LC-3 in PC12 and CL1-0 cells in response to Compound C treatment. Cell lysates were collected at 8 hr post ALA-PDT for LC-3 western blot. β-actin was used as an internal control.

During autophagy, the microtubule-associated protein-1 light chain 3 (LC3) is processed from an 18 kDa LC3-I form to a 16 kDa LC3-II form [35]. Therefore, increased level of LC3-II is an additional characteristic of autophagy due to its association with the autophagosome membrane. To further assess the involvement of autophagy after ALA-PDT, we performed immunoblot analysis to detect the formation of LC3-II. Consistent with the MDC staining and the punctuated LC3-GFP signals, elevated amounts of LC3-II were observed in ALA-PDT-treated PC12 cells (Fig. 4B, left panel). The increased LC3-II protein level was also found in the CL1-0 cell line after ALA-PDT (Fig. 4B, right panel). Moreover, addition of the autophagy inhibitor, 3-methyladenine (3-MA), increased the PC12 cell survival rate in a dose-dependent manner after ALA-PDT (Fig. 5A, left panel). Similar results were also found in CL1-0 cells (Fig. 5B, left panel). Meanwhile, ALA-PDT-induced autophagy contributed to a portion of the activation of caspase-9 and -3 as 3-MA could partially decrease the activation of these caspases (right panels of Fig. 5A &5B). Taken together, despite the possible co-existence of multiple cell death fates, these results indicate that autophagy is the major cell death pathway in PC12 and CL1-0 cells following ALA-PDT.

### AMPK, but not JNK nor p38, mediates autophagic cell death following ALA-PDT

We have demonstrated that the activation of AMPK was associated with ALA-PDT-induced cell death (Fig. 2). To investigate whether AMPK activation mediates ALA-PDT-induced autophagic cell death, the expression level of autophagic marker LC3-II was examined in the absence or presence of Compound C. As shown in Fig. 5C, Compound C inhibited the formation of LC3-II in both PC12 and CL1-0 cells treated with ALA-PDT, indicating that ALA-PDT induced autophagic cell death is mediated by AMPK.

**Figure 6:**
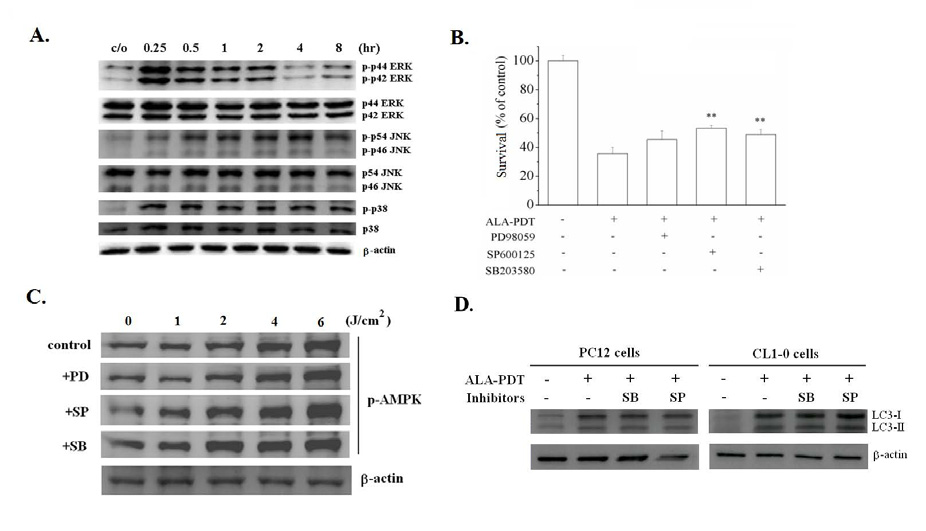
**Activation of JNK and p38 involves in the ALA-PDT mediated cell death but not relates to the AMPK activation and autophagic cell death**. (**A**) PC12 and CL1-0 cells were treated with 1 mM ALA for 3 hr, and then irradiated under the light dose of 2 and 12 J/cm^2^, respectively. The cell lysates were collected at the time indicated after ALA-PDT. Kinase activity and protein level of the three MAPKs were analyzed by western blot analysis with specific anti-phospho- or anti-MAPK antibodies. β-actin was also used as an internal control. Representative results from at least three independent experiments are shown. (**B**) PC12 cells were subjected ALA-PDT with or without the inhibitors of the three MAPKs. The blockers applied were PD98059 (ERK inhibitor, 40 μM), SP600125 (JNK inhibitor, 40 μM), and SB203580 (p38 inhibitor, 40 μM). Cell survival was analyzed 24 hr post ALA-PDT. Significantly different from cells treated with ALA-PDT only; **P < 0.01. (**C**) PC12 cells were subjected ALA-PDT in the presence of the three MAPKs inhibitors. Cell lysates were collected 30 minutes post ALA-PDT (1 mM ALA, 2 J/cm^2^). AMPK activity was analyzed by western blot analysis using antibody recognized the p-AMPK. (**D**) In the presence of the inhibitor of JNK or p38, PC12 and CL1-0 cells were treated with 1 mM ALA for 3 hr, and then irradiated under the light dose of 2 and 12 J/cm^2^, respectively. Cell lysates were collected at 8 hr post ALA-PDT and further used for LC-3 immunoblot analysis.

Activations of JNK and p38 kinases have been reported in human HaCaT keratinocytes and hypopharyngeal carcinoma FaDu cells treated with ALA-PDT [36,37]. In agreement with previous findings, we found that ERK, JNK, and p38 were activated in response to ALA-PDT treatment, while the total level of these protein kinases remained constant (Fig. 6A). The rapid activation of the three MAPKs was found as early as 15 minutes after light irradiation. Furthermore, JNK and p38 but not ERK played a role in mediating ALA-PDT cytotoxicity because JNK and p38 inhibitors significantly increased cell survival rate in ALA-PDT-treated PC12 cells (Fig. 6B).

**Figure 7:**
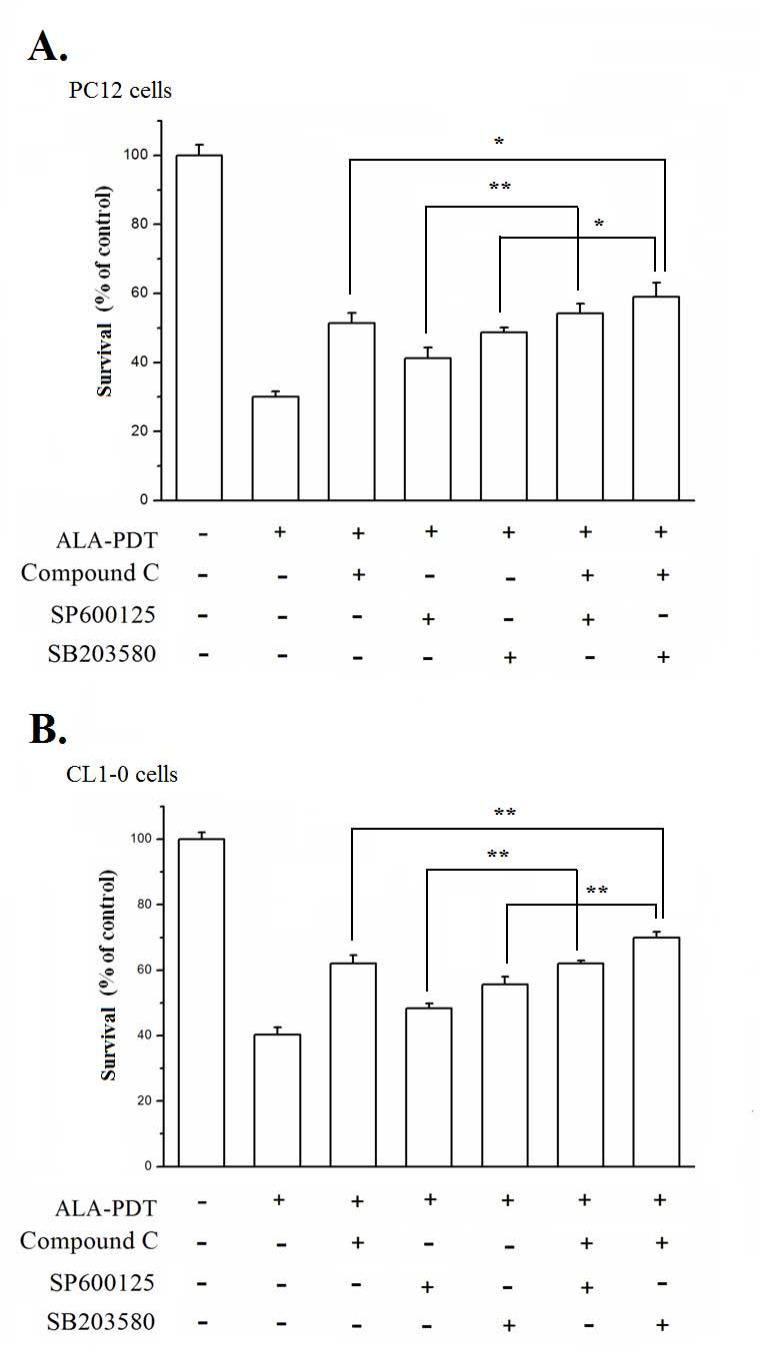
**Effects of the combined AMPK and MAPK inhibitors on PC12 and CL1-0 cell survival after ALA-PDT**. In the presence of AMPK and MAPK inhibitors, PC12 and CL1-0 cells were treated with 1 mM ALA for 3 hr and then irradiated under the light dose of 2 and 12 J/cm^2^, respectively. Cell survival was analyzed by Trypan Blue exclusion assay 24 hr post ALA-PDT. Effects of AMPK (Compound C, 40 μM), JNK (SP600125, 40 μM) and p38 (SB203580, 40 μM) inhibitors on ALA-PDT induced cell death were analyzed in PC12 (**A**) and CL1-0 (**B**) cells. Results are representative of three independent experiments and are shown as mean ± SD. Significantly different from ALA-PDT with Compound C, SP or SB; *P < 0.05, **P < 0.01.

As activation of MAPKs precedes the AMPK activation, we then examined whether MAPKs were involved in the activation of AMPK by determining the level of phosphorylated AMPK in the presence of MAPK inhibitors. As shown in Fig. 6C, blocking the three MAPK kinases did not affect the AMPK activation, suggesting that the elevated activity of AMPK is not related to MAPKs activation. Furthermore, the formation of LC3-II after ALA-PDT was not interfered with by JNK and p38 inhibitors (Fig. 6D), indicating that the ALA-PDT-induced autophagic cell death was not a consequence of MAPKs activation in PC12 and CL1-0 cells. Finally, treatment with both AMPK and p38 inhibitors exhibited an additive effect in preventing cell death as compared to the individual inhibitor alone in PC12 (Fig. 7A) and CL1-0 cells (Fig. 7B). This suggests that the activation of AMPK and p38 are two separated pathways stimulated by ALA-PDT. Together, these data demonstrate for the first time that the activation of AMPK is meditating autophagic cell death in cells treated with ALA-PDT.

## Discussion

The commitment events as well as the modality of cell death induced by PDT have been extensively studied. It is widely agreed that the action mechanism of PDT is largely dependent on (1) the preferential subcellular localization of the photosensitizer, (2) the molecular targets of the photosensitizer, and (3) the genotype of the cell. Although caspase-dependent apoptosis has been documented as the predominant cell death modality after PDT [5,29,38-40], recent studies have also demonstrated that PDT may induce non-apoptotic cytotoxicity via the induction of autophagic cell death pathway [24,41]. In this study, we observed that PC12 and CL1-0 cells underwent autophagy after ALA-PDT as indicated by multiple independent approaches that either revealed the formation of autophagic vacuoles or the expression of autophagy specific proteins (Fig. 4). In addition, the cell death was prevented by the autophagy inhibitor but not the caspase antagonist (Fig. 5). These results led to our conclusion that autophagy is the predominant form of cell death in both PC12 and CL1-0 cells in response to ALA-PDT. However, it should be noted that the autophagy inhibitor (3-MA) could not fully rescue the ALA-PDT-induced cell death and also partially decreased the activity of caspase-9 and -3 (Fig. 5A and 5B, right panel). These observations might relate to the loss of mitochondrial membrane potential (MMP) induced by ALA-PDT. It is possible that subsequent to the loss of MMP, cytochrome c released from mitochondria might still induce the activation of caspase-9 and caspase-3, although caspase activation in this fashion did not play a role in ALA-PDT mediated cell death. In addition, other cell death regulators, such as apoptosis-inducing factor (AIF) and the endonuclease G might be released from the mitochondria as a result of MMP breakdown. These molecules could further lead to caspase-independent DNA fragmentation [42], which explains the oligonucleosomal DNA fragmentation induced by ALA-PDT (Fig. 3A). This notion could be further supported by the findings of Furre *et al*. [39] that showed that PpIX-mediated mitochondrion-photosensitization induces apoptosis through translocation of AIF in human leukemia cells. In this regard, we still cannot rule out the possibility that, other than autophagy, ALA-PDT might induce other modes of caspase-independent cell death in PC12 and CL1-0 cells. Further investigations are required to fully characterize the concomitant mediators and determinant switches of other minor types of cell death that might coexist in PC12 and CL1-0 cells in response to ALA-PDT.

Although PDT-induced stress-activated pathways linked to cell death have been explored widely, direct biochemical associations between the stress signals and cell death, especially autophagy, remain poorly characterized. Activation of the three MAPKs has been described in various cell lines following PDT. However, the role of AMPK had not been reported in PDT biology. In an attempt to identify the major molecular event(s) required for autophagic cell death induced by ALA-PDT, we evaluated the activity of AMPK and MAPK protein kinase families in PC12 and CL1-0 cells. We found that the activation of AMPK and JNK and p38 was one of the earliest molecular events involved in the ALA-PDT-induced cell death. Significant activation of JNK and p38 was found to precede the activation of AMPK after ALA-PDT in both PC12 and CL1-0 cell lines. Nevertheless, AMPK and MAPK are independent effectors of ALA-PDT because further studies showed that the level of AMPK activation remained unaffected by the MAPK blockers (Fig. 6C). Vice versa, inhibition of AMPK did not alter the activation level of MAPKs induced by ALA-PDT in PC12 and CL1-0 cells (data not shown).

Although the activation of MAPK signaling could induce autophagy [43], MAPKs are not involved in mediating autophagic cell death in ALA-PDT treated cells because the formation of autophagy specific LC3-II protein remained unaltered in response to the treatment of JNK and p38 inhibitors (Fig. 6D). Conversely, the AMPK inhibitor could effectively prevent the cell death after ALA-PDT and could reduce the expression level of autophagy-associated LC3-II protein. These results together provide a novel yet intriguing link between AMPK signaling cascade and autophagic cell death triggered by ALA-PDT. Based on these results, we conclude that autophagic cell death induced by ALA-PDT requires the activation of AMPK but not MAPKs in both PC12 and CL1-0 cells. Although JNK and p38 are not involved in the ALA-PDT-induced autophagic cell death (Fig. 6B), it should be noted that the combination of AMPK and p38 inhibitors has additive effect in increasing the cell survival after ALA-PDT compared to the AMPK or p38 inhibitor alone (Fig. 7). Together, these data further demonstrate that MAPKs and AMPK are independent pathways involved in ALA-PDT-induced cell death.

Our investigations reported in this article have revealed the novel association of the activation of AMPK to ALA-PDT-induced autophagic cell death. These findings shed light on the development of new anticancer therapeutic modality. However, the complex picture of multiple signaling cascades that link PDT-induced oxidative stress to the ultimate autophagic cell death remains far from complete. Further characterization of the intermediating mechanisms may reveal novel therapeutic strategy in anti-cancer treatment.

## Conclusion

In this study, we found that ALA-PDT effectively induced oxidative stress that led to immediate mitochondrial dysfunction, and finally resulted in a caspase-independent cell death in PC12 and CL1-0 cells. In response to ALA-PDT, activation of AMPK, JNK, and p38 signaling cascades were found to promote cell death. However, it was the activation of AMPK but not JNK or p38 that mediated autophagic cell death in PC12 and CL1-0 cells. These results indicate the involvement of AMPK and p38 pathways in this complex mechanism.

## Materials and methods

### Chemical reagents and antibodies

ALA, N-acetyl-L-cysteine (NAC), and monodansylcadaverine (MDC) and 3-methyladenine (3-MA) were purchased from Sigma (St. Luis, MO, USA). 5,5',6,6'-tetrachloro-1,1',3,3'- tetraethylbenzimida- zolylcarbocyanine iodide (JC-1) and ATP determination kit were purchased from Molecular Probes (Eugene, OR, USA). Lipofectamine 2000 transfection kit and Trypan Blue were purchased from Invitrogen (Carlsbad, CA, USA). Antibodies to phosphorylated ERK, p38, and AMPK or total ERK, JNK and p38 were purchased from Cell Signaling Technology (Danvers, MA, USA). Anti-phospho-JNK antibody, Caspase-3/CPP32 and Caspase-9 fluorometric assay kits were purchased from BioVision (Mountain View, CA, USA). Monoclonal antibody that recognizes both forms of LC3 was purchased from MBL International Cooperation (Woburn, MA, USA). PD98059 and SB203580 were purchased from Promega. SP600125, Compound C and zVAD-fmk (Z-Val-Ala-Asp(OMe)-fluoromethyl ketone) were obtained from Calbiochem (San Diego, CA, USA).

### Cell culture, transfection, and photodynamic treatment

PC-12 cells were incubated in DMEM supplemented with 5% fetal bovine serum (FBS) and 10% heat-inactivated horse serum (HS). CL1-0 cells were derived from a poorly differentiated human lung adenocarcinoma [28] and cultured in RPMI medium containing 10% FBS. Cells were cultured at 37°C in a humidified atmosphere containing 5% CO_2_. The GFP-LC3 transfected PC12 cells were produced by transfecting pGFP-LC3 plasmid into subconfluent PC12 cells using Lipofectamine 2000 transfection kit. For ALA-PDT, cells were seeded onto culture dishes or chamber slides and grown overnight in complete medium. Unless otherwise specified, cells were then incubated with 1 mM ALA for 3 hr, and then exposed to various doses of light. The light source is consisted of high power LED array, with the wavelength centered at 635 ± 5 nm [25]. The light dose is 1-6 J/cm^2 ^(PC12) or 12 J/cm^2 ^(CL1-0) at an intensity of 60 mW/cm^2^. After irradiation, cells were incubated in complete medium until further analyzed.

### Cell survival rate

After ALA-PDT, cellular cytotoxicity was determined by Trypan Blue exclusion assay. Briefly, cells were trypsinized 24 hr post ALA-PDT and co-incubated with 0.4% (w/v) Trypan Blue at room temperature for 10 min. Cells with Trypan blue uptake were counted as dead cells on a hemacytometer. Cells exposed to ALA without light irradiation were used as controls. Each individual phototoxic experiment was repeated for three times.

### Analysis of mitochondrial membrane potential

Prior to examine the mitochondrial membrane potential, cells were seeded into chamber slides and allow growing for 24 hr. On the next day, cells were incubated with 1 mM ALA for 3 hr. For the last 20 min of ALA incubation, cells were stained with 10 μg/ml of JC-1 for the examination of mitochondrial membrane potential. After light irradiation, JC-1 staining patterns were visualized with the confocal spectral microscope (Leica, model TCS SP2). JC-1 was excited by a 488 nm agron-ion laser and the emitted fluorescence was measured at 525 ± 20 nm for JC-1 monomers and 615 ± 25 nm for JC-1 aggregates. All experiments were performed under ambient light.

### Determination of mitochondrial ATP content

Mitochondrial ATP content was measured with an ATP determination kit according to manufacturer's instructions (Molecular Probes, Eugene, OR, USA). Briefly, cells were plated and incubated in glucose-free culture medium for 3 hr to avoid the production of ATP via glycolysis. At the time indicated after ALA-PDT, cells were lysed with 1% Triton and mixed with ATP reaction buffer before measuring the luminescence by a luminescence counter. The relative luminescence intensities were corrected with the amount of total protein in the cell extract. Three measurements were taken from each sample for statistical analysis.

### Assays of DNA fragmentation and caspase enzymatic activity

For DNA fragmentation analysis, PC12 cells were returned to the complete medium after ALA-PDT and cell pellets were collected at the time indicated. DNA from the cell pellets was isolated with QIAamp DNA Mini kit according to the manufacturer's instructions (Qiagen, Valencia, CA, USA) before further analyzed in a 1.5% agarose gel. After electrophoresis, the gel was stained with 0.5 μg/ml ethidium bromide and photographed under UV light. For caspase activity, PC12 & CL1-0 cells were seeded into Petri-dishes. After ALA-PDT, cells were returned to the complete medium and cell lysates were collected at the time indicated to determine the caspase-9 and -3 like activities by the assay kit used according to manufacturer's instructions.

### Fluorescence imaging of MDC and GFP-LC3

PC12 or GFP-LC3 transfected PC12 cells were seeded into Chamber Slides and incubated for 24 hr. On the next day, cells were treated with ALA-PDT. At the time indicated, PC12 cells were stained with 0.05 mM MDC in PBS for the imaging of autophagosomes. To detect the re-distribution of GFP-LC3 after ALA-PDT, GFP-LC3 transfected PC12 cells were fixed and co-stained with Hoechst 33342 to label the nucleus. Cells were then washed with PBS and immediately observed under microscope. The MDC fluorescence images were taken under Zeiss Axiophot 2 Fluorescence Microscope. The fluorescence photographs of LC3-GFP were recorded upon excitation by a 488 nm agron-ion laser and the emission measured at 525 ± 25 nm using the confocal fluorescence microscope (Leica TCS SP5). A Diode UV laser (405 nm) was used to excite Hoechst 33342 and the emitted fluorescence was collected at 445 ± 15 nm.

### Immunoblot analysis

Cells treated with ALA-PDT were lysed at the time indicated. Equal amount of protein lysates were electrophoresed on 10% SDS-polyacrylamide gels. The proteins were transferred to the nitrocellulose membrane and incubated with antibodies recognizing both form of LC-3, phosphorylated form of ERKs, JNK, p38, AMPK proteins or β-actin (as loading control). We also determined the total amount of ERK, JNK and P38 using antibodies specific to all forms of ERK, JNK and p38, respectively. Secondary antibody conjugated with horseradish peroxidase was applied and immunocomplex was visualized by Chemiluminescence Reagent Plus (Blossom Biotechnology Inc., Boston, USA).

### Statistical analysis

All experiments were repeated at least three times with 4~6 parallel measurements in different dishes or slides. Results were averaged from experiments performed under the same conditions and described as the mean ± SD unless stated otherwise. The statistical significance of differences in the results was analyzed using Student's *t*-test for paired experiment or one-way ANOVA test for multiple comparisons.

## Competing interests

The authors declare that they have no competing interests.

## Authors' contributions

HTJ participated in the design of the experiment and carried out the work. LTC participated in the design of the study and coordinated the draft of the manuscript. YHL participated in the design of cell death analysis and carried out the work. HFC participated in the design of the study and performed the statistical analysis. HTJ, LTC, YHL, and HFC read and approved the final manuscript. CTC conceived the study, participated in its design and coordination and finalized the draft of the manuscript. All authors have read and approved this manuscript.
